# Young Astrocytic Mitochondria Attenuate the Elevated Level of CCL11 in the Aged Mice, Contributing to Cognitive Function Improvement

**DOI:** 10.3390/ijms24065187

**Published:** 2023-03-08

**Authors:** Ryosuke Tashiro, Dan Ozaki, Jesus Bautista-Garrido, Guanghua Sun, Lidiya Obertas, Alexis S. Mobley, Gab Seok Kim, Jaroslaw Aronowski, Joo Eun Jung

**Affiliations:** Department of Neurology, McGovern Medical School, University of Texas Health Science Center at Houston, Houston, TX 77030, USA

**Keywords:** aging, cognition, hippocampus, astrocytes, mitochondria, CCL11, neuroplasticity

## Abstract

Aging drives cognitive decline, and mitochondrial dysfunction is a hallmark of age-induced neurodegeneration. Recently, we demonstrated that astrocytes secrete functional mitochondria (Mt), which help adjacent cells to resist damage and promote repair after neurological injuries. However, the relationship between age-dependent changes in astrocytic Mt function and cognitive decline remains poorly understood. Here, we established that aged astrocytes secret less functional Mt compared to young astrocytes. We found the aging factor C-C motif chemokine 11 (CCL11) is elevated in the hippocampus of aged mice, and that its level is reduced upon systemic administration of young Mt, in vivo. Aged mice receiving young Mt, but not aged Mt improved cognitive function and hippocampal integrity. Using a CCL11-induced aging-like model in vitro, we found that astrocytic Mt protect hippocampal neurons and enhance a regenerative environment through upregulating synaptogenesis-related gene expression and anti-oxidants that were suppressed by CCL11. Moreover, the inhibition of CCL11-specific receptor C-C chemokine receptor 3 (CCR3) boosted the expression of synaptogenesis-related genes in the cultured hippocampal neurons and restored the neurite outgrowth. This study suggests that young astrocytic Mt can preserve cognitive function in the CCL11-mediated aging brain by promoting neuronal survival and neuroplasticity in the hippocampus.

## 1. Introduction

Aging drives cognitive impairment and is a non-modifiable risk factor for many cerebrovascular and neurodegenerative diseases [1,2,3,4,5]. The hippocampus is central to many aspects of cognitive functions and is particularly susceptible to neurodegeneration during aging [5,6,7,8,9,10,11]. The aged hippocampus shows decreased synaptic plasticity and downregulation of plasticity-related genes [12,13], suppression of neurogenesis [14,15,16,17], and antioxidant imbalance [18,19], which together result in progressive cognitive decline [1,5,6,7,8,9,15]. 

Growing evidence suggests that exposure to young circulatory factors helps to rejuvenate impaired functions in aged tissues [15,20,21,22,23,24,25]. However, cognitive decline is one of the major aspects of aging, and the development of approaches that could preserve cognitive integrity is in much demand as the elderly proportion is growing [26,27]. C-C motif chemokine 11 (CCL11) is known as “blood-circulating aging factor,” which plays a key mediating role in age-related cognitive decline [15]. The levels of CCL11 are increased with aging in both humans and mice, and CCL11 is associated with impaired cerebral function, including learning and memory [15,22,28,29,30]. CCL11 rapidly crosses the blood–brain barrier (BBB), suggesting that blood-borne CCL11 may directly affect the brain [20,31] and cognitive decline [14,15,16,17,20,31]. The CCL11-main receptor is C-C chemokine receptor 3 (CCR3), which is highly expressed by microglia and hippocampal neurons [12,13,32,33]. Villeda and colleagues revealed that the peripheral plasma level of CCL11 was correlated with impaired cognitive functions in aged mice [15]. Using a heterochronic parabiosis mouse model, the authors demonstrated that CCL11 present in the systemic milieu inhibits adult neurogenesis in the hippocampus, and affects synaptic plasticity in an age-dependent manner [15]. In addition, systemic exposure to CCL11 induces impairment of learning and memory in young adult mice [15]. It was further demonstrated that systemic neutralization of CCL11, using neutralizing antibodies to CCL11, improves cognitive functions in aged mice [15]. 

In an aging brain, oxidative phosphorylation (OXPHOS) and ATP production in mitochondria (Mt) are decreased [34,35]. Mitochondrial dysfunction, such as MtDNA mutation, reactive oxygen species (ROS) overproduction, and loss of anti-oxidative capacity [36,37,38,39], is a hallmark of age-related neurodegeneration in the human and mouse [40,41,42]. Thus, restoration of mitochondrial function could help to slow down aging-induced neurodegeneration. Emerging data in rodent and human revealed that Mt can transfer between cells in the brain to assist the recipient cells in improved functioning, and to avert injury, suggesting that the use of healthy functional Mt as a therapeutic approach for brain rejuvenation deserves investigation [43,44,45,46,47,48]. Hayakawa and his colleagues demonstrated that astrocytes in the brain secrete functional Mt into adjacent neurons to help neuronal survival by increasing ATP levels and recovery after ischemic stroke [47]. Recently, we demonstrated that astrocytes secrete functional Mt that can be taken up by microglia and neurons, where they promote a “healing phenotype” of microglia and increase neuronal connectivity through mechanisms involving the upregulation of anti-oxidative manganese superoxide dismutase (Mn-SOD) and production of small mitochondria peptide, humanin [48,49]. During aging, synaptic mitochondrial dysfunction in the hippocampus causing ROS overproduction, respiration rate decrease, and reduction in ATP generation contributes to memory loss and cognitive impairment [50]. In addition, mitochondrial dysfunction during aging results in impaired synaptic neurotransmission and hippocampal synaptic plasticity that induces impairments in learning and memory [51]. Mt damages caused by aging could also impair hippocampal neurogenesis, which could promote cognitive dysfunction [52,53,54,55,56,57,58]. 

In this study, we hypothesize that the transfer of young astrocytic Mt can rejuvenate the hippocampal synaptic plasticity and cognitive function in the CCL11-governed aging brain. We aim to investigate the causality between aging-related deterioration of astrocytic Mt and cognitive decline in the aging brain. First, we demonstrated the different functionality and “health” of astrocytic Mt released from young and aged adult astrocytes. Second, we demonstrated the rejuvenating capacity of young astrocytic Mt in the hippocampus under a CCL11-induced aging environment, which coincided with improved cognitive function in aged mice.

## 2. Results

### 2.1. Aged Astrocytes Release Less Functional Mt Compared to Young Astrocytes in Culture

We and others previously demonstrated that astrocytes secrete functional Mt, which, upon entering adjacent cells, helps them to resist damage and promotes repair after neurological injuries [47,48,49]. The extracellularly released Mt from the cultured astrocytes from rat or mouse brains are viable and functional, and exert pro-survival effects and enhance anti-oxidant capacity [47,48,49]. However, the age-dependent change in Mt functionality by astrocytes and its effect on cognitive decline in the aged brain remains poorly understood. Here, we examined if extracellularly secreted Mt from aged astrocytes shows impaired functionality compared to Mt from young astrocytes. 

First, we cultured primary adult astrocytes from young (3-month-old) and aged (22-month-old) mice brains, with minor modifications to the previously reported method [59]. To verify the purity of astrocytes, we performed immunocytochemistry and Western blot by using GFAP, vimentin, ALDH1L1, and EAAT-2 as astrocyte markers (Supplemental Figure S1B,C). Immunostaining confirmed an intense cytoplasmic immunolabeling of GFAP (on the date of fourth passages), vimentin, and ALDH1L1 (on the date of eighth passages) in the aged astrocytes that were cultured from 22-month-old mice brains, attesting to the astrocytic phenotype of the cultured cells (Supplemental Figure S1B). Western blot also detected EAAT-2, vimentin, and ALDH1L1 proteins in cell lysates from both 3-month-old (young) and 22-month-old (aged) adult astrocytes (Supplemental Figure S1C, on the date of eighth passages). Next, we assessed the levels of cellular senescence in young (3-month-old) and aged (22-month-old) astrocytes by β-galactosidase assay, using FACS analysis. We found that β-galactosidase-positive astrocytes were more abundant in aged astrocytes (~78.6%) compared with young astrocytes (~30.0%) (Supplemental Figure S1D). 

**Figure 1:**
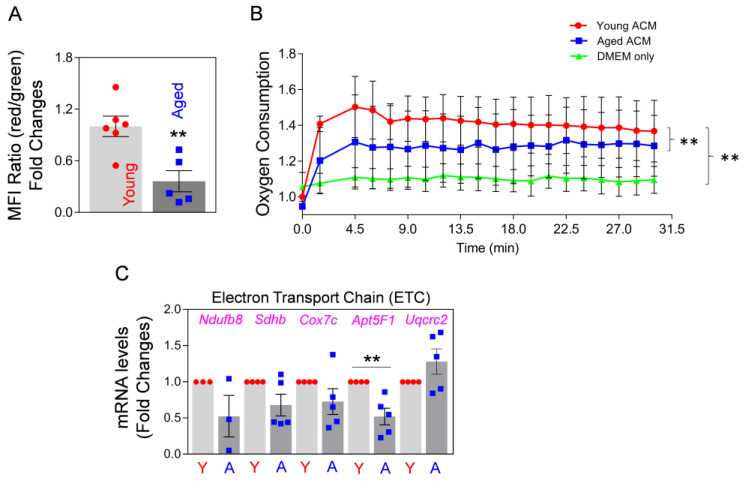
Aged cultured astrocytes release less functional Mt compared to young astrocytes. (**A**) FACS analysis using JC-1 staining, showing membrane potential of Mt released from the cultured young and aged astrocytes harvested from 3-month-old vs. 22-month-old mice brains, respectively. Young or aged astrocytes in culture were incubated with 0.2 µM JC-1 for 30 min. After washing with PBS three times, the astrocytes were incubated with fresh culture media for 24 h to allow accumulation of extracellularly released Mt. The membrane potential of Mt released into astrocyte-conditioned medium (ACM) was measured by FACS. To evaluate Mt membrane potential, the ratio of red/green fluorescence was calculated (Mt functionality index); the ratio is proportional to membrane potential, where a high ratio indicates high membrane potential and high functionality of Mt. Data represent fold changes in mean fluorescence intensity (MFI) ratio of red/green fluorescence. The significance was assessed by a two-tailed unpaired *t*-test (*n* = 5–6 per group): ** *p* < 0.01 (*p* = 0.005, young vs. aged ACM), *t* value (*t* = 3.695). Values are shown as mean ± SEM. (**B**) Real-time oxygen consumption in young and aged Mt in ACM from the cultured young and aged astrocytes, respectively. Astrocyte culture media only (20% FBS in DMEM) served as the negative control. The fluorescence was measured after adding 20 μL O_2_ consumption reagent to 300μL ACM or media every 1.5 min up to 30 min. Data represent mean ± SEM. The difference among groups was determined by Repeated Measure One-way ANOVA with Tukey’s multiple comparison test (*n* = 3 per group): ** *p* < 0.01 (*p* < 0.0001, young vs. aged ACM), *q* value (*q* = 18.17); ** *p* < 0.01 (*p* < 0.0001, young ACM vs. DMEM only), *q* value (*q* = 21.25). (**C**) mRNA expression analysis for electron transport chain (ETC) components (*Ndufb8, Sdhb, Cox7c, Apt5F1*, and *Uqcrc2*) by qRT-PCR in cultured young and aged astrocytes. Data represent mean ± SEM. The difference among groups was determined by unpaired *t*-test (*n* = 3–5 per group): ** *p* < 0.01 (*p* = 0.0078, young vs. aged astrocytes in *Apt5F1*), *t* value (*t* = 3.684).

Secondly, we investigated Mt function by measuring the membrane potential of the extracellularly-secreted Mt in astrocyte-conditioned medium (ACM), which were collected after a 24 h incubation of young (3-month-old) and aged (22-month-old) astrocytes after JC-1 staining in culture. We found a significant reduction in membrane potential in the Mt from aged astrocytes compared with Mt from young astrocytes (Figure 1A). Next, we measured oxygen consumption of Mt in each ACM derived from young and aged astrocytes. Oxygen consumption of Mt from aged astrocytes was significantly lower than in the Mt from young astrocytes (Figure 1B). These data suggest that the function of Mt released from aged astrocytes is compromised. To compare the function of intracellular Mt regarding oxidative phosphorylation (OXPHOS) between young and aged astrocytes, we measured the levels of mRNA expression of gene products involved in the electron transport chain (ETC). Among five ETC constituents, the mRNA expression levels of Ndufb8, Sdhb, Cox7c, and Apt5F1 were markedly lower in aged astrocytes compared with young astrocytes (Figure 1C). Collectively, our results suggest that as astrocytes age, they may lose Mt function regarding membrane potential and oxidative respiration. Moreover, our data clearly show that the Mt released from aged astrocytes are functionally compromised, as compared to the Mt released from young astrocytes.

### 2.2. Circulating Level of Aging Factor CCL11 Is Increased in Aged Hippocampus, and the Effect of That Could Be Reversed with Young Astrocytic Mt along with Improved Cognitive Function and Hippocampal Integrity

**Figure 2:**
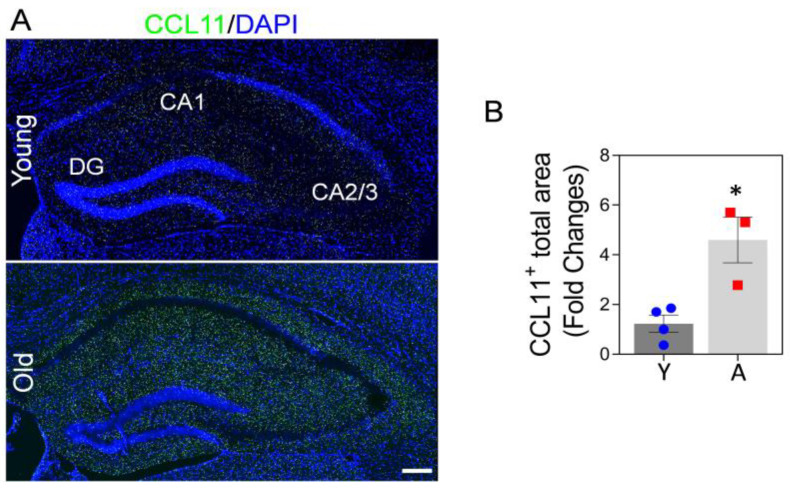
CCL11 is highly elevated in the hippocampal area of aged mice brains. (**A**) Representative immunofluorescence confocal images of CCL11 (green) staining in the hippocampus of young (3-month-old) vs. aged (22-month-old) mice brains. Images indicate higher level of CCL11 in the aged hippocampus compared to the young hippocampus. Nuclei were stained with DAPI (blue). Scale bar, 200 µm. (**B**) The illustrated graph shows a significant difference in CCL11- positive areas. N = 3–4 animals per group. The significance was assessed by a two-tailed unpaired *t*-test: * *p* < 0.05 (*p* = 0.0116, young vs. aged mice), *t* value (*t* = 3.881). All data are shown as mean ± SEM. Y, young; A, aged.

Circulating CCL11 increases with age, which may lead to oxidative stress, neuronal death, impaired neurogenesis, synaptic loss, and learning and memory deficits in aged mice and elderly humans [15,25,60]. CCL11 is a blood-circulating factor, which can cross the BBB [20]. We found that the levels of CCL11 in the hippocampus of 22-month-old aged mice are higher compared to the hippocampus of 3-month-old mice (Figure 2A,B). This may suggest that high CCL11 in the hippocampus of aged mice could affect hippocampal neurons and cognitive function. It was recently demonstrated that astrocytic Mt enhance neuroplasticity through energy supply, pro-survival effects, and anti-oxidant defense capacity in neurons [47,49]. Here, we hypothesize that systemic administration of young and functional astrocytic Mt can modulate the adverse effect of CCL11 on hippocampus function in the aged animal.

**Figure 3:**
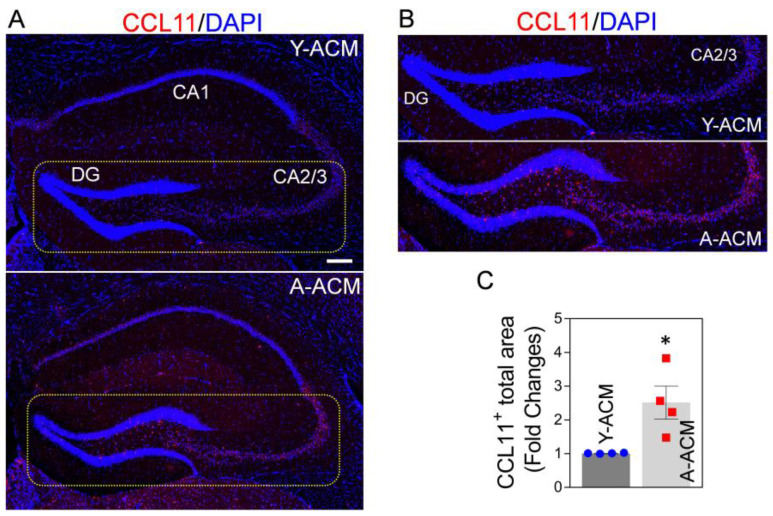
Elevated CCL11 in aged hippocampus is attenuated upon administration of young astrocytic Mt. (**A**) CCL11 (red) representative immunohistochemistry staining in the hippocampus of the aged (22-month-old) mice intravenously receiving the ACM containing young Mt (Y-ACM) or the ACM containing aged Mt (A-ACM) collected from young or aged astrocytes cultured from 3-month or 22-month-old mice brains, respectively. The animal received Y-ACM or A-ACM once a week for 4 weeks. Nuclei were stained with DAPI (blue). Scale bar, 200 µm. (**B**) Magnified confocal images from the yellow boxes in Figure 3A demonstrate that the highly elevated CCL11 in the dentate gyrus (DG) and cornu ammonis (CA)2/3 areas of the aged hippocampus were diminished with Y-ACM. (**C**) The illustrated quantifying graph shows a difference in CCL11 immunofluorescence signal positive areas in the hippocampus of the aged mice that received Y-ACM vs. A-ACM. N = 4 animals per group. The significance was assessed by a two-tailed unpaired *t*-test: * *p* < 0.05 (*p* = 0.0215, Y-ACM vs. A-ACM), *t* value (*t* = 3.087). All data are shown as mean ± SEM.

First, we tested if systemic administration of young astrocytic Mt could lower CCL11 levels in the hippocampus of aged mice. We found that the intravenous injection of ACM containing young astrocytic Mt (Y-ACM) to aged mice significantly reduced the age-elevated hippocampal CCL11 (Figure 3A,C), while administration of ACM containing aged Mt (A-ACM) failed to reduce CCL11 levels. This effect was especially robust in the dentate gyrus (DG) and cornu ammonis (CA)2/3 areas (Figure 3B, magnified images from the indicated yellow boxes of Figure 3A). We also showed that the ACM containing astrocytic Mt subjected to filtration (0.22 µm pore column) to remove Mt had no effect on the CCL11 levels, confirming the essential role of Mt in this process (Supplemental Figure S2A). In addition to central nervous system (CNS) effects, administration of young astrocytic Mt markedly attenuated the level of CCL11 in the plasma of aged mice (Supplemental Figure S2B).

**Figure 4:**
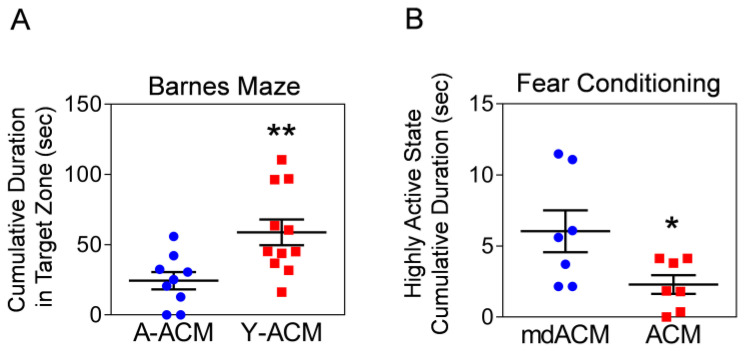
Systemically administered young astrocytic Mt improve the cognitive function of aged mice. (**A**) 22-month-old mice intravenously received Mt-containing ACM from astrocytes generated from either young (3-month-old; Y-ACM) or aged (22-month-old; A-ACM) mice brain once a week for 4 weeks. At 4 weeks, mice were tested in the Barnes maze (spatial learning and memory test). Mice receiving young astrocytic Mt (Y-ACM) demonstrated significantly improved performance (increased time spent in the target zone) compared to animals that received aged Mt (A-ACM). The significance was assessed by a two-tailed unpaired t-test (*n* = 9–11 per group): ** *p* < 0.01 (*p* = 0.0082, A-ACM vs. Y-ACM), *t* value (*t* = 2.97). All data are shown as mean ± SEM. (**B**) 22-month-old mice received either ACM or Mt depleted ACM (mdACM; filtered ACM) once a week for 4 weeks. The mice were subjected to a fear conditioning (associative learning) test. Mice receiving astrocytic Mt have a significant decrease in cumulative time of highly active state (meaning increase in cumulative freezing time) in response to the light cue, compared to animals receiving mdACM. The significance was assessed by a two-tailed unpaired *t*-test (*n* = 7 per group): * *p* < 0.05 (*p* = 0.0383, ACM vs. mdACM), *t* value (*t* = 2.326). All data are shown as mean ± SEM.

Next, we investigated if treatment of aged mice with young astrocytic Mt can also improve the cognitive deficit of these animals. Twenty-two-month-old mice once a week for 4 weeks intravenously received ACM containing Mt from cultures of astrocytes generated from either young (3-month-old) or aged (22-month-old) mice. At 4 weeks, the animals were tested for spatial learning and memory using Barnes maze. The mice receiving young astrocytic Mt showed significantly better performance (increased time spent in the target zone) compared to the mice receiving aged Mt (Figure 4A). In the subsequent experiment, 22-month-old mice were injected once a week for 4 weeks with ACM or Mt-depleted ACM (filtered to remove Mt) prior to being tested using the fear conditioning (associative learning) test. The mice receiving Mt showed a significant decrease in cumulative time of the highly active state (meaning increase in cumulative freezing time) in response to the cue (light), compared to the animals that received Mt-depleted fraction (mdACM) (Figure 4B). To investigate if this improvement in cognitive function in aged mice was due to improved hippocampal integrity, we measured the NeuN-positive neurons by using immunohistochemistry in the hippocampus of aged mice administered Y-ACM vs. A-ACM. We found the increases in NeuN-positive cells in the DG, CA1, and CA2/3 areas of the hippocampus in the aged mice that received Y-ACM compared to the mice that received A-ACM (Supplemental Figure S3). Our results suggest that systemic administration of young astrocytic Mt to aged animals reduces levels of hippocampal CCL11, while improving hippocampal integrity and cognitive functions.

### 2.3. Astrocytic Mt Improves Neuronal Viability and Synaptic Structure in CCL11-Induced Aging-Like Model, In Vitro

**Figure 5:**
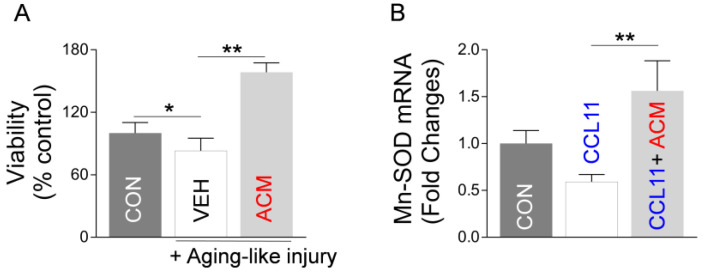
Astrocytic Mt enhance viability and antioxidant enzyme expression in hippocampal neurons under CCL11-induced aging-like environment in vitro. (**A**) Viability of hippocampal neurons under CCL11-induced aging-like injury (50 ng/mL CCL11 with 70 μΜ H_2_O_2_) with treatment of ACM. Viability was evaluated by lactate dehydrogenase (LDH) assay. Data represent mean ± SEM. The difference among groups was determined by one-way ANOVA with Tukey’s multiple comparisons (*n* = 4–7 per group). * *p* < 0.05 (*p* = 0.0263 control vs. aging-like injury), *q* value (*q* = 4.145); ** *p* < 0.01 (*p* < 0.001 vehicle vs. ACM in aging-like injury), *q* value (*q* = 15.82). (**B**) mRNA level (qRT-PCR) of Mn-SOD in hippocampal neurons treated with ACM or vehicle, followed by exposure to 50 ng/mL CCL11. Data represent mean ± SEM. The difference among groups was determined by one-way ANOVA with Tukey’s multiple comparisons (*n* = 5–6 per group). ** *p* < 0.01 (*p* = 0.0081 vehicle vs. ACM in CCL11 treatment), *q* value (*q* = 5.052).

**Figure 6:**
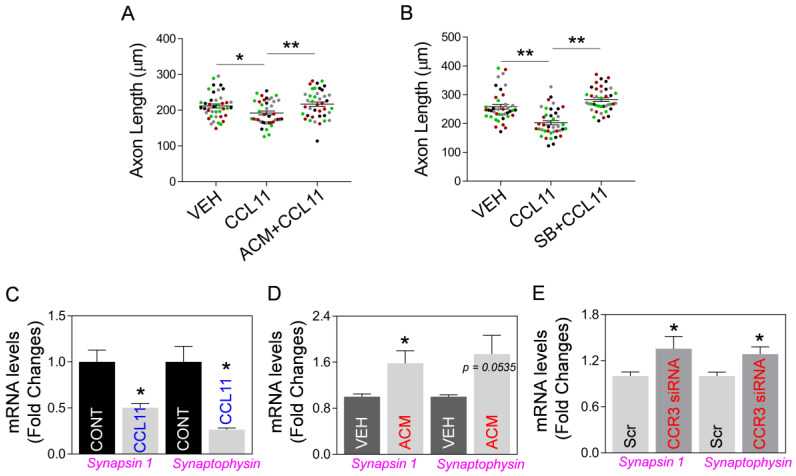
Astrocytic Mt preserve neurite outgrowth and the expression of synaptogenesis-related genes in primary neurons under CCL11-induced aging-like environment through CCR3, in vitro. (**A**,**B**) Quantitation of neurite outgrowth in rat primary cultured neurons pre-treated with (**A**) ACM in cortical neurons or (**B**) 10 µM SB328437 (SB; CCR3 inhibitor) in hippocampal neurons, followed by exposure to 50 ng/mL CCL11. Media alone or DMSO (0.01%) were used as vehicle controls. Ten individual neurites in culture from each treatment group were manually traced, and the length of each neurite was measured using ImageJ software. The significant differences in neurite length changes were assessed by one-way ANOVA/Fisher’s LSD multiple comparisons test (four colors represent one of four independent experiments, *n* = 4): Data are shown as mean ± SEM, and the mean was calculated by averaging ten values for each experiment. (**A**) * *p* < 0.05 (*p* = 0.0292 vehicle vs. CCL11), *t* value (*t* = 2.591); ** *p* < 0.01 (*p* = 0.0097 CCL11 vs. ACM + CCL11), *t* value (*t* = 3.267). (**B**) ** *p* < 0.01 (*p* = 0.0004 vehicle vs. CCL11), *t* value (*t* = 5.409); ** *p* < 0.01 (*p* < 0.0001 CCL11 vs. SB328437 + CCL11), *t* value (*t* = 7.84). (**C**) mRNA levels of synapsin 1 and synaptophysin in the hippocampal neurons treated with 50 ng/mL CCL11 or vehicle. Data represent mean ± SEM. The difference among groups was determined by unpaired *t*-test (*n* = 3 per group). * *p* < 0.05 (*p* = 0.0220 vehicle vs. CCL11), *t* value (*t* = 3.64) in synapsin 1; * *p* < 0.05 (*p* = 0.0123 vehicle vs. CCL11), *t* value (*t* = 4.331) in synaptophysin. (**D**) mRNA levels of synapsin 1 and synaptophysin in the hippocampal neurons treated with ACM or vehicle under CCL11 treatment. Data represent mean ± SEM. The difference among groups was determined by unpaired *t*-test (*n* = 5 per group). * *p* < 0.05 (*p* = 0.0301 vehicle vs. ACM), *t* value (*t* = 2.632) in synapsin 1; *p* = 0.0535 vehicle vs. ACM in synaptophysin. (**E**) mRNA levels of synapsin 1 and synaptophysin in the cultured hippocampal neurons transfected with scrambled (Scr)- or CCR3-specific siRNA. Data represent mean ± SEM. The difference among groups was determined by unpaired t-test (*n* = 5–8 per group). * *p* < 0.05 (*p* = 0.0462 scrambled- vs. CCR3 siRNA), *t* value (*t* = 2.311) in synapsin 1; * *p* < 0.05 (*p* = 0.0168 scrambled- vs. CCR3 siRNA), *t* value (*t* = 2.743) in synaptophysin.

Next, using an aging-like in vitro model based on exposure of neurons to aging factor CCL11, we examined if astrocytic Mt could indeed improve hippocampal neuron integrity in this model. Thus, we developed an in vitro model of CCL11-induced aging-like injury by exposing cultured primary hippocampal neurons to a combination of a low dose of CCL11 (50 ng/mL) and a sublethal level of hydrogen peroxide (70 μM). The neuronal viability was decreased by aging-like injury and this injury was reversed by ACM treatment (Figure 5A). In addition, while analyzing CCL11-treated hippocampal neurons, we found that CCL11-induced reduction of Mt-specific anti-oxidant Mn-SOD mRNA is reversed by astrocytic Mt (Figure 5B). Next, we examined if astrocytic Mt enhances neuronal outgrowth in the primary cultured neurons under the CCL11-induced aging-like environment. We measured the length of neurites in cultured hippocampal or cortical neurons after exposing them to various modifying conditions (CCL11, astrocytic Mt-ACM, and SB328437-CCR3 inhibitor). CCL11 alone significantly inhibited neurite outgrowth, which was effectively restored by astrocytic Mt (Figure 6A). The CCL11-specific receptor CCR3 is highly expressed by hippocampal neurons [12,13,32]. Treatment with a CCR3 inhibitor also restored neurite extension deficit inhibited by CCL11 (Figure 6B), suggesting that CCL11 inhibits neurite extension via its receptor CCR3. Next, to better understand the nature of neuronal modification, we examined if astrocytic Mt enhance the expression of synaptogenesis-related genes under a CCL11-induced aging-like environment in vitro. CCL11 significantly attenuated the expression of synaptogenesis-related genes such as synapsin 1 and synaptophysin in the cultured hippocampal neurons (Figure 6C). The hippocampal neurons exposed to astrocytic Mt under CCL11 treatment showed restoration in mRNA levels of synapsin 1 and synaptophysin (Figure 6D). Furthermore, we found that the knockdown of CCR3 by using CCR3-specific siRNA enhanced the expression levels of synaptogenesis-related genes (Figure 6E). Collectively, our data suggest that the CCL11-induced aging-like environment attenuates neuronal viability and neuroplasticity in the hippocampal neurons in vitro; however, astrocytic Mt can reverse these CCL11-induced inhibitory effects through enhancing gene expression of anti-oxidant defense and synaptogenesis.

## 3. Discussion

Effective approaches helping to alleviate cognitive decline and the process of aging are needed as the world’s elderly population is growing [26,27]. Several experiments, including heterochronic parabiosis, suggested new promising strategies to slow down the aging process, including cognitive decline [15,22,61,62,63,64,65]. The hippocampus is central to many aspects of cognitive functions [5,6,7,8,9]. The aged hippocampus shows decreased size, reduced synaptic plasticity, downregulation of plasticity-related genes [12,13], suppression of neurogenesis [14,15,16,17], and antioxidant imbalance [18,19], which together result in progressive cognitive decline. 

CC-motif chemokine 11 (CCL11) is a member of the eotaxin family [29,66] that is robustly increased in the circulation in aged humans and mice [15,28], and its elevated level is implicated in the loss of cognitive function and an increase in age-induced neurodegeneration [15,60,67]. CCL11 freely crosses the blood–brain barrier (BBB) [15,20,28] and, by acting through the CC-motif chemokine receptor 3 (CCR3), triggers neurodegenerative processes such as oxidative stress, synaptic disruption, and inhibition of neurogenesis [15,17,24,62]. Systemic administration of CCL11 impairs learning and memory in young animals by impairing hippocampal function and adult neurogenesis [15]. In this study, we found that the level of CCL11 is highly elevated in the aged hippocampus, not only in the plasma. CCL11 signals primarily through CCR3 which is abundant in neurons [12,13,32]. Our study indicates that the CCL11/CCR3 signaling pathway is indeed involved in the neuronal vulnerability and that signaling through this pathway weakens synaptic plasticity associated with aging in the hippocampus. We found that CCL11 attenuated the mRNA level of the mitochondrial anti-oxidant enzyme manganese superoxide dismutase (Mn-SOD) in cultured hippocampal neurons, suggesting a mechanism responsible for weakening anti-oxidative defense and increasing neuron vulnerabilities to injury. Moreover, CCL11 decreased the neurite outgrowth in hippocampal cultured neurons that coincided with the reduction of mRNA expression of synaptic-related genes such as synapsin 1 and synaptophysin. Importantly, the knockdown of CCR3 in the cultured hippocampal neurons boosted mRNA levels in these synaptic-related genes. Our data indicate that the CCL11/CCR3 pathway could indeed represent a central target to prevent hippocampal neurons from the aging-induced loss of hippocampal integrity. 

While our present study does not focus on the role of CCL11/CCR3 signaling in adult hippocampal neurogenesis, it is important to stress that neurogenesis is one of the key factors that maintains hippocampal integrity and cognitive functions. In the aged hippocampus, the loss of neural stem cell (NSC) production was shown in the dentate gyrus (DG) [68,69,70] region of the hippocampus, with strong neurogenic activities and roles in cognitive functions [71]. Our ongoing study found that CCL11 inhibits neuronal differentiation of adult hippocampal NSCs in vitro, suggesting that CCL11 can act as a factor inhibiting adult hippocampal neurogenesis in the aged brain. 

There have been some reports showing that CCL11 reduction by experimental challenges in aged mice restored cognitive function [15,72,73]. Recently, we demonstrated that astrocytes release functional Mt which can be taken up by microglia and neurons, where they promote a “healing” phenotype in microglia and enhance the anti-oxidative capacity of neurons, contributing to improved functional recovery from brain injury [48,49]. Our earlier study also demonstrated that MitoTracker-labeled astrocytic Mt were abundantly seen in the soma and neurite of cultured primary neurons after their transfer for at least 7 days [49]. Emerging data also suggest that Mt transfer between cells in the brain is to assist the recipient cells in improved functioning, suggesting that the use of healthy functional Mt as a therapeutic approach for aged brain rejuvenation deserves further investigation [43,44,45,46,47,48,49]. Mitochondrial dysfunction is a hallmark of age-induced neurodegeneration [40,41,42,56]. However, the mechanism by which dysfunctional Mt in the brain influences the aging-mediated cognitive decline is not clear. Our present study showed that the functionality of Mt released from aged vs. young astrocytes is compromised. We found that the membrane potential, oxygen consumption rate, and mRNA levels of the electron transport chain (ETC) of aged astrocytes are impaired. 

**Figure 7:**
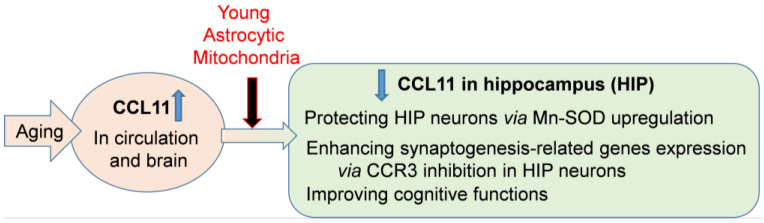
A graphical abstract summarizing the results. Systemic administration of young astrocytic Mt can improve cognitive functions in the CCL11-mediated brain of aged mice by protecting hippocampal neurons and enhancing synaptogenesis-related gene expression.

We demonstrated here that systemic transfer of young astrocytic Mt into aged mice attenuates the level of CCL11 in the hippocampus as well as in the plasma of aged mice, and that these Mt improve the cognitive function of aged mice and enhance hippocampal integrity (Figure 7). This may be associated with a neuroprotective effect of Mt, as we also demonstrated that astrocytic Mt have neuroprotective capacity in ameliorating the CCL11-induced and oxidative stress-induced injury to hippocampal neurons, in vitro. Mt robustly prevented the reduction in Mn-SOD level caused by CCL11, which otherwise could result in defective defense from oxidative damage. An additional benefit of astrocytic Mt is that they can restore neuroplasticity inhibited by CCL11/CCR3, through upregulation of synaptogenesis-related gene expression and neurite outgrowth. Although this study proposes the CCL11/CCR3 axis as a major signaling pathway involved in the young astrocytic Mt-mediated cognition improvement in aged animals, other pathways may also in parallel contribute to this improvement. One possible mechanism is neuroprotective signaling mediated by the Mt-generated small peptide, humanin (HN). HN is an Mt-encoded small peptide and an Mt-secreted molecule with neuroprotective effects [48,49]. In our earlier studies, HN promoted the “reparative” phenotype of microglia by enhancing the peroxisome proliferator-activated receptor gamma (PPARγ) transcriptional pathway in the cultured microglia [48]. Also, HN enhanced anti-oxidative capacity and synaptic plasticity in the cultured neurons against intracerebral hemorrhagic-like injury [49]. These earlier studies suggest that HN is one of the key molecules in the astrocytic Mt-regulated neuroprotection. Others also demonstrated that the astrocyte-released HN prevented synaptic loss in the hippocampal neurons, suggesting that a reduction in HN with age may represent a possible mechanism for synaptic dysfunction and cognitive decline [74].

There are many remaining questions, and one is how can the functional astrocytic Mt decrease the levels of CCL11 both in the brain and plasma of aged mice? Blood-circulating CCL11 is elevated with aging in humans and rodents; however, the main cellular sources of CCL11 are still not clear. Earlier studies reported peripheral organs, epithelial cells, fibroblasts, smooth muscle cells, and tissue-resident macrophages as generating CCL11 [75,76,77,78]. CCL11 could be also produced by astrocytes, microglia, pericytes, and choroid plexus epithelial cells in the central nervous system under inflammation conditions [24,79,80]. In this study, astrocytic Mt were administered into the aged mice via the femoral vein, attenuating both plasma and brain CCL11 levels. Thus, it remains unclear whether the systemically administered astrocytic Mt interact with the cells in the peripheral organs upon entering the circulation, thereby leading to the reduction in CCL11 production, or if Mt directly enter the brain parenchyma where they interact with brain cells such as astrocytes or microglia, contributing to CCL11 reduction in the aged brains. 

In addition, there are some limitations in the current study. Although this study showed that systemic administration of young ACM improved cognitive function in aged mice when compared to administration of aged ACM, it is also possible that systemic administration of aged ACM may lead to cognitive decline. In the current study, we directly compare the efficacy of young ACM vs. aged ACM in enhancing cognitive function, since the functionality of astrocytic Mt in the ACM was decreased with age in our in vitro study. However, the possibility exists that aged or unhealthy astrocytes (e.g., under disease conditions) could secrete harmful factors, which could attenuate cerebral function, including cognition, compared to young and healthy astrocytes [81,82]. Moreover, damaged Mt due to aging could lead to overproduction of reactive oxygen species (ROS) that can interfere with the regulation of cellular homeostasis in the brain [36,37,38,39]. Thus, future study on whether the aged astrocytes or aged astrocytic Mt generate any detrimental factors that can drive cognitive decline in the aged brain is required to fully elucidate the molecular mechanism of age-dependent cognitive decline.

Collectively, our study shows the interaction between age-dependent deterioration of astrocytic Mt and hippocampal degeneration. This study suggests a potential translational value of using young astrocytic Mt for hippocampal rejuvenation and cognitive improvement in the aged brain. We demonstrate that healthy and functional astrocytic Mt are capable of maintaining hippocampal integrity and improving cognitive function, probably by promoting hippocampal neuron health, and by enhancing neuroplasticity through mitigating the adverse role of CCL11 in the aging environment. 

## 4. Materials and Methods

### 4.1. Animals

Three-month-old and twenty-month-old C57BL6/J male or female mice were obtained from Jackson Laboratories (Bar Harbor, ME, USA) and the National Institute of Health (NIH). Pregnant female Sprague Dawley rats were purchased from Charles River Laboratories (Wilmington, MA, USA) for primary cell cultures. All animals were randomly assigned to each experiment. All animal experimental procedures were conducted in compliance with the NIH guideline Animal Care and Use of Laboratory Animals and approved by the Animal Welfare Committee of the University of Texas Health Science Center in Houston.

### 4.2. Primary Astrocyte Cultures from Adult Mice Brains or Rat Embryonic Brains

Primary astrocyte cultures were performed using 3-month-old young and 22-month-old aged adult mice with minor modifications from the previously reported method [59]. The cortices were dissected from each brain by carefully removing the meninges, superficial vessels, striatum, hippocampus, and olfactory bulb. The cortices were minced and enzymatically digested by using 5 mL of 0.25% Trypsin at 37 °C for 15 min with gentle mixing every 5 min. After, cells were passed through a 100 µm pore mesh, and single-cell suspensions were made by mechanical dissociation and plated onto poly-D-lysine-coated 75-cm^2^ flasks and cultured in Dulbecco’s medium essential medium (DMEM) supplemented with 20% fetal bovine serum (FBS), 50 µg/mL streptomycin, 50 U/mL penicillin, 10 µM forskolin (Stock concentration: 10 mM in dimethyl sulfoxide) (F3917, Sigma), and 10 ng/mL glial cell-derived neurotrophic factor (GDNF) (Stock concentration: 10 µg/mL in distilled water) (21-8505, TONBO Biosciences, San Diego, CA). Astrocytes were cultured in 37 °C humidified incubators with 5% CO_2_. After seeding, the adherent cells formed astrocyte colonies on the coated bottom of the culture flask in the next several days and gradually started to spread on the coated bottom in the next couple of weeks. The medium was changed every 3–4 days. After growing, the cultured cells gave rise to astrocyte-enriched cultures in three to four weeks (Supplemental Figure S1A, phase-contrast images of aged astrocytes cultured from 22-month-old mice brains; D-10 or D-100 after seeding). Once the astrocytes in the culture flask reached ~80% confluence, the cells could be trypsinized and re-plated onto the new culture flask to expand the cell populations. The cell culture passages were possible up to 7–8 times. Primary rat astrocyte culture was prepared using embryonic day 17 Sprague Dawley rat brains with our well-established protocol [48,49]. Briefly, the cortices were digested by using 0.25% trypsin and suspended as a single-cell solution and seeded onto poly-D-lysine-coated 75-cm^2^ flasks. The mixed glial cells were cultured in DMEM with 10% FBS, 50 µg/mL streptomycin, and 50 U/mL penicillin. Once confluent, the cells were re-plated onto a new flask by using the trypsin method and subjected to shaking to remove microglia from the cell layer. After discarding floating cells, the pure astrocyte monolayer was incubated with new fresh culture media.

### 4.3. Primary Neuron Cultures

Primary hippocampal or cortical neurons were isolated from the embryonic day 17 Sprague Dawley rat brains, as described in our previous protocol with minor modification [49]. Hippocampus or cortices were collected by dissecting the brain to remove the meninges, striatum, and olfactory bulb. The tissues were subject to mechanical and enzymatic digestion with 0.25% trypsin for 10 min. Single cell suspension was plated onto poly-D-lysine-coated plates and cultured in DMEM which included 10% FBS, 50 µg/mL streptomycin, and 50 U/mL penicillin for the first 24 h. On the next day, the culture medium was changed to Neurobasal medium containing 0.5 mmol/L glutamine, 2% B-27, and 1% antibiotic–antimycotic. The culture medium was changed every 2–3 days. At DIV6-7, the neurons were used for further experiments.

### 4.4. Preparation of Astrocyte-Conditioned Medium (ACM)

To collect the extracellularly released mitochondria (Mt) from adult mouse astrocytes (young vs. aged astrocytes), astrocyte-conditioned medium (ACM) from each cultured astrocyte was prepared as reported previously [47,48,49]. Shortly, 7 mL of fresh culture media was supplied to astrocyte cultures in a 75 cm^2^ flask. After 24 h of incubation, the culture media was collected and centrifuged at 1000 rpm for 5 min to remove cellular debris. The supernatant containing extracellularly-released Mt from astrocytes was collected as Y-ACM (ACM containing young Mt from young astrocytes) and A-ACM (ACM containing aged Mt from aged astrocytes). Then, 200 µL Y-ACM or A-ACM per injection was intravenously administrated (i.v. injections once a week for 4 weeks) via the femoral vein of mice under general anesthesia using isoflurane. In addition, to collect the extracellularly released Mt from rat primary astrocytes cultured from rat E17 brains, the ACM from the cultured rat astrocytes was also prepared as reported previously [47,48,49]. To remove Mt from the ACM, the mdACM (Mt-depleted ACM) was obtained by filtration of the ACM through a 0.22 µm filter, as described in our previous well-established protocols [48,49]. 200 µL ACM or mdACM per injection was intravenously administrated (i.v. injections once a week for 4 weeks) via the femoral vein of mice under general anesthesia using isoflurane.

### 4.5. Measurement of Mitochondrial Membrane Potential

To measure the functionality of released Mt from cultured astrocytes, mitochondrial membrane potential was assessed by using the cyanine dye 5,5′,6,6′-tetrachloro-1,1′,3,3′-tetraethylbenzimidazolylcarbocyanine iodide (JC-1, T-3168, Invitrogen) following the previous study [47]. Briefly, the cultured astrocytes were treated with 0.2 μM JC-1 and incubated at 37 °C for 30 min. After PBS washing three times and replacement with fresh media, the astrocytes were cultured for 24 h to allow accumulation of JC-1-labelled Mt. The fluorescences from green (Ex/Em = 485 nm/516 nm) and red (Ex/Em = 579 nm/599 nm) lasers were analyzed using a Cytoflex S cytometer (Beckman Coulter). The ratio of red/green fluorescence was regarded as an index to estimate the low and high mitochondrial membrane potential. All data were analyzed using FlowJo software ver.10.7.1.

### 4.6. Detection of Cellular Senescence in Adult Astrocytes

To measure cellular senescence in the cultured adult astrocytes by detecting β-galactosidase hydrolysis, CellEvent Senescence Green Flow Cytometry Assay (C10840, ThermoFisher) was performed following the manufacturer’s guidelines. Briefly, the adult astrocytes were fixed by using 2% paraformaldehyde (PFA) in PBS, stained with CellEvent Senescence Green Probe for 90 min in a 37 °C incubator with no CO_2_, washed with PBS containing 1% BSA, suspended in 1% bovine serum albumin (BSA) in PBS, and then analyzed on a flow cytometer using 488 nm laser and 530 nm/30 filter. 

### 4.7. Immunocytochemistry

Astrocytes in culture were subject to fixation with 4% PFA for 15 min at room temperature, permeabilization with 0.3% Triton-X in PBS for 30 min at room temperature, and incubation with blocking buffer containing 3% BSA, 10% goat serum, and 0.1% Triton-X for 3 h at room temperature. After washing with PBS three times, astrocytes were incubated with primary antibodies against glial fibrillary acidic protein (GFAP) (1:200 dilution, ab16997, Abcam), vimentin (1:200 dilution, ab92547, Abcam), and aldehyde dehydrogenase 1 family member L1 (ALDH1L1) (1:200 dilution, ab87117, Abcam) overnight in 4 °C. After washing three times, astrocytes were subject to 2 h incubation with either Alexa 488- or Alexa 568-conjugated secondary anti-rabbit antibodies (1:500 dilution) at room temperature. Images were visualized using Zeiss LSM 800 confocal microscope (Carl Zeiss, Germany).

### 4.8. Immunohistochemistry

All procedures for immunohistochemistry were conducted in accordance with our established protocols [48,49]. Shortly after, mice were anesthetized and underwent cardiac perfusion with cold PBS and 4% PFA. After fixation with 4% PFA for 24 h, the brain tissues were dehydrated by 30% sucrose for 24 h. Brain sections (thickness 20 or 30 μm) were prepared using a microtome (HM450, Thermo Scientific) and preserved in an anti-freezing solution (30% ethylene glycerol, 30% glycerol, 20 mM sodium phosphate, and 20 mM sodium hydroxide in distilled water) at −20 °C until further processing. Brain sections were subject to permeabilization with 0.3% Triton-X in PBS for 30 min at room temperature and incubation with blocking buffer (0.1% Triton X, 5% goat or horse serum, and 3% BSA in PBS) for 1 h at room temperature. Then, sections were incubated with primary antibodies against anti-neuronal nuclei (NeuN), Alexa 488-conjugated antibody (1:200 dilution, MAB377X, Millipore), or CCL11 (1:200 dilution, AF-420-NA, R&D) at 4 °C for 24 h. After washing with PBS for 10 min three times, sections were incubated with Alexa FluorTM Plus 488 donkey anti-goat antibody (1:500 dilution, A32814, Invitrogen) or Alexa FluorTM Plus 546 donkey anti-goat antibody (1:500 dilution, A11056, Invitrogen) for 2 h at room temperature. Nuclei were stained with DAPI in PBS at room temperature for 10 min. After mounting with Prolong Diamond Antifade (P36970, Invitrogen), images were visualized using Zeiss LSM 780 confocal laser scanning microscope (Carl-Zeiss, Germany).

### 4.9. Measurement of Real-Time Oxygen Consumption

Oxygen consumption of astrocytic Mt in young ACM, aged ACM, and the negative control (regular astrocyte culture medium) was measured by an extracellular O_2_ consumption kit (ab197243, Abcam), as previously described [47,49]. The regular adult astrocyte culture medium (DMEM with 20% FBS, 50 µg/mL streptomycin, and 50 U/mL penicillin) was used as the negative control. Briefly, 20 μL O_2_ consumption reagent was added to each well containing 300 µL young or aged ACM and gently mixed. The fluorescence was continuously measured every 1.5 min up to 30 min with the filter combination of Excitation = 380 nm/Emission = 650 nm.

### 4.10. Measurement of Cell Viability

The viability of primary hippocampal neurons in vitro was determined by measuring lactate dehydrogenase (LDH) activity with a Cytotoxicity Detection kit (Roche Applied Science) as described previously [49]. Shortly, primary hippocampal neurons (3.0 × 10^5^ neurons/well) were treated with ACM for 24 h, followed by exposure to 50 ng/mL CCL11 (NBP2-35294, NovusBio) and 70 μM hydrogen peroxide for 24 h. Neuronal cell lysis was prepared by treating with 0.1% Triton-X for 15 min and centrifuging at 14,000 rpm for 15 min. After incubation with LDH reaction buffer, the absorbance at 490 nm was measured. Cell viability was calculated based on the amount of remaining LDH in the cytosolic sample. 

### 4.11. CCR3 Knockdown Using siRNA

Primary rat hippocampal neurons were seeded in 12-well plates at 3.0 × 10^5^ cells/well. siRNA knockdown targeted rat CCR3 using FlexiTube GeneSoultion GS117027 for Ccr3 (1027416, QIAGEN). Hippocampal neurons were transfected with 10 nmol/L by using HiPerFect siRNA Transfection reagent (301704, QIAGEN) following the manufacturer’s instructions. Scrambled siRNA (SIC001, Sigma) was used as the negative control.

### 4.12. Real-Time Quantitative RT-PCR

Total RNA was isolated from the cultured mouse adult astrocytes or rat hippocampal neurons using RNeasy Mini kit (QIAGEN). Then, cDNA was synthesized using amfiRivert Platinum One cDNA synthesis Platinum Master Mix (R6100, GenDEPOT, Katy, TX, USA) according to the manufacturer’s instructions. Real-time quantitative PCR (RT-qPCR) was conducted by using amfiSure qGreen Q-PCR Master Mix without ROX (Q5600, GenDEPOT, Katy, TX, USA). The following primer sets were used: Mouse NADH dehydrogenase (Ndufb8) Fw, 5′- ATCAGTGGGACCACTCAGAA-3′; Mouse Ndufb8 Rv, 5′-AAAACCATGAAAGCCACAAA-3′; Mouse succinate dehydrogenase complex subunit B (Sdhb) Fw, 5′- TAAGTGCGGACCTATGGTGT-3′; Mouse Sdhb Rv, 5′- TTGGAGACTTTGCTGAGGTC -3; Mouse ubiquinol cytochrome c reductase core protein 2 (Uqcrc2) Fw, 5′- ACATATCAAAAGGGGCAACA -3′; Mouse Uqcrc2 Rv, 5′- GCAACTGCTTTGACTTGGTT-3′; Mouse cytochrome c oxidase subunit 7c (Cox7c) Fw, 5′- AAGGGGAGTTAGGTGGTACG -3′; Mouse Cox7c Rv, 5′- TTCCACTGAAAATGGCAAAT -3′; Mouse ATP synthase peripheral stalk-membrane subunit b (Atp5pb) Fw, 5′- AAAAGCATGTGGTGAAGAGC -3′; Mouse Atp5pb Rv, 5′- CTGAGCTTGAGCCTTCTTTG -3′; Rat Mn-SOD Fw, 5′-AAGCGTGACTTTGGGTCTTT-3′; Rat Mn-SOD Rv, 5′-ATCCCCAGCAGTGGAATAAG-3′; Rat Synapsin1 Fw, 5′- GTGTCAGGGAACTGGAAGACC -3′; Rat Synapsin1 Rv, 5′- AGGAGCCCACCACCTCAATA -3′; Rat Synaptophysin Fw, 5′- ACTACTCCTCGTCGGCTGAA -3′; Rat Synaptophysin Rv, 5′-ACAGGGTCCCTCAGTTCCTT -3′; Rat GAPDH Fw, 5′- AGA CAG CCG CAT TTC TTG T -3′; Rat GAPDH Rv, 5′- CTT GCC GTG GTA GAG TCA T -3′.

### 4.13. Western Blot

Adult mouse astrocytes in culture were lysed using RIPA buffer which included halt protease and phosphatase inhibitor cocktail (catalogue no. 78443, Thermo Fisher Scientific), followed by centrifugation at 14,000× *g* at 4 °C for 15 min. Then, 30 μg of protein was mixed with Laemmli sample buffer (NP0007, Invitrogen) at 90 °C for 5 min. Proteins were separated on 4–20% gradient Tris-Glycine polyacrylamide gels (Thermo Fisher Scientific), followed by transfer onto nitrocellulose membranes by the iBlot2 Dry Blotting System (Thermo Fisher Scientific). Membranes were incubated with blocking buffer (927-6001, LI-COR), followed by incubation with primary antibodies against excitatory amino acid transporter 2 (EAAT-2) (ab41621, Abcam), vimentin (ab92547, Abcam), and aldehyde dehydrogenase 1 family member L1 (ALDH1L1) (ab87117, Abcam) at 1:1000 dilution at 4 °C overnight at room temperature. After washing with 0.5% Tween-20 TBS, the membranes were incubated with IRDye^®^ 800CW donkey anti-rabbit IgG (926-32213, LI-COR) secondary antibody at 1:2000 dilution for 1 h. Images were captured by LI-COR ODYSSEY CLx. 

### 4.14. Assessment of Neurite Outgrowth

Neurite outgrowth in primary cultured neurons was evaluated as described previously [49]. Rat primary neurons were seeded onto poly-D-lysine-coated 12-well plates. The next day after seeding (DIV 1), the immature neurons were treated with or without ACM for 24 h, and then treated with 50 ng/mL CCL11 for 6 days. For the preparation of ACM to treat the immature neurons for in vitro neurite outgrowth experiments, the culture media of astrocytes were replaced with Neurobasal media containing 2% B-27, 0.5 mmol/L-glutamine, and 1% antibiotic–antimycotic 24 h prior to ACM treatment. Also, the immature neurons (DIV 1) were treated with vehicle vs. 10 µM SB328437 (CCR3 inhibitor, 3650, R&D) 1 h prior to CCL11 treatment and cultured for 6 days. At DIV7, phase-contrast images were captured with an Olympus IX81 motorized inverted microscope (Olympus, Tokyo, Japan). Individual neurite lengths from each group were manually traced, and their lengths were analyzed using ImageJ software. 

### 4.15. BioPlex Luminex Immunoassay

The detection and quantification of mouse cytokines from small amounts of mouse plasma samples were performed using Bio-Plex Pro Mouse Cytokine 23-plex Assay (M60009RDPD, Bio-Rad) according to the manufacturer’s instructions. Briefly, the 10× coupled beads were diluted to 1× in Bio-Plex Assay Buffer and transferred to each well of the assay plate. After washing the plate with Bio-Plex Wash Buffer, 50 µL of plasma samples, standards, and blank were added to each well. After incubating the plate on a shaker at room temperature, the diluted 1× detection antibodies were added to each well. After incubation with antibodies, each well was washed, and 1× streptavidin-PE was added to each well and incubated on a shaker. After resuspending in 125µL assay buffer, the plate was read using the setting for the Bio-Plex100, 200, and 3D Systems and instrument settings for the Bio-Plex MAGPIX System (Bio-Rad) following the manufacturer’s instructions. 

### 4.16. Assessment of Cognitive Function

The Barnes maze test [83,84] and fear conditioning test [85,86] with modifications were used to assess spatial and associative learning and memory in mice, respectively. Briefly, the Barnes maze test was performed using an elevated circular platform that had 20 holes evenly spaced. An escape box was equipped under one of the holes, which allowed the mouse to hide in a dark space. Support frames with visual cues surrounding the platform, bright lights, and an overhead camera were equipped in the testing room. Each behavior was traced/recorded and analyzed using Noldus EthoVision XT 14 behavior software (Leesburg, Virginia). The procedure for an entire test was composed of 3 training trials (1st trial: 3 min habituation in a start chamber, 2nd trial: 30 s habituation in a start chamber, exploring the platform for 2 min, and 3rd trial: repeat of 2nd trial) and a test trial (30 s habituation in a start chamber, exploring the platform for 3 min). The time taken to get into the escape box under the target hole or the cumulative duration in the target zone that had an escape box were measured. The fear conditioning test was performed using a rectangular chamber with a chamber light and electric shock. Mouse behavior inside the chamber was also traced/recorded and analyzed using Noldus EthoVision XT 14 behavior software. The procedure for an entire test was composed of a training phase (2 min light only) and a shock phase (2 min light followed by 0.5 mA shock for 2 s) on the first day, and a testing phase (3 min light only) on the second day. The cumulative duration of a highly inactive or active state was measured. 

### 4.17. Statistical Analysis

Animals for in vivo experiments were randomly assigned to each treatment group. All the data analysis was conducted using Graph Pad Prism 10.7.1 software (GraphPad Software Inc., San Diego, CA, USA). One-way analysis of variance (ANOVA) with Tukey’s or Fisher’s LSD method was applied when comparing multiple groups. An unpaired t-test was applied when comparing two groups. Repeated Measure (RM) One-way ANOVA with Tukey’s multiple comparison test was applied when comparing the difference in groups from the oxygen consumption assay. All the analyses were two-sided. Data represent mean ± standard error medium (SEM). *p*-values below 0.05 were regarded as statistically significant. 

## Data Availability

All data generated or analyzed for this study are included in this published article.

## Supplementary Materials

The following supporting information can be downloaded at https://www.mdpi.com/article/10.3390/ijms24065187/s1.
