# Supplementary material for: Young Astrocytic Mitochondria Attenuate the Elevated Level of CCL11 in the Aged Mice, Contributing to Cognitive Function Improvement
Source: Int J Mol Sci. 2023 Mar 8;24(6):5187. doi: 10.3390/ijms24065187 (PMC10049211; doi:10.3390/ijms24065187)
Supplement: Supplementary file 1 [file ijms-24-05187-s001.zip › ijms-2259574-supplementary.pdf]

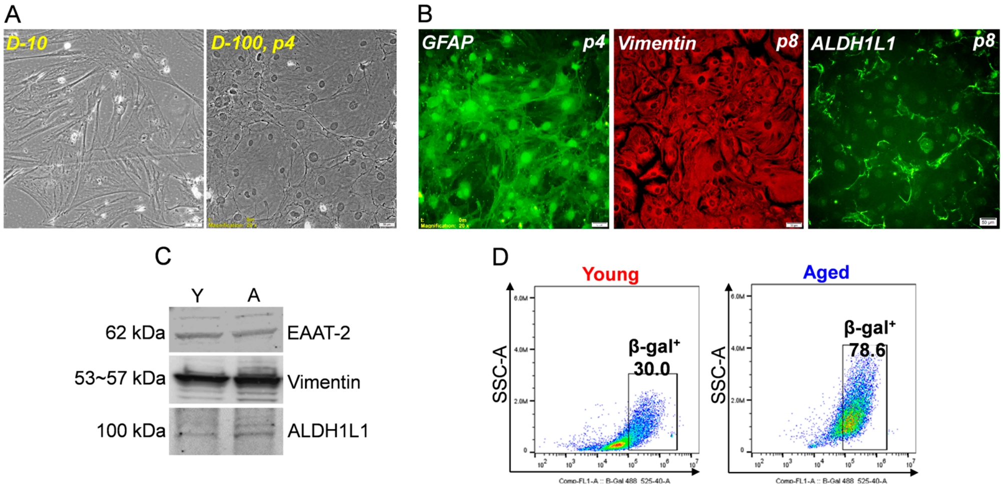

Supplementary Figure S1. Primary adult astrocyte cultures using young and aged mice brains. (**A**) Two phase-contrast images showing the morphology of cultured astrocytes at D-10 or D-100 (on 4th passages, p4), respectively, after seeding from 22-month-old aged mice brains. Scale bar, 50 μm. (**B**) Three immunofluorescent images by using astrocyte markers such as GFAP (on 4th passages), vimentin, and ALDH1L1 (on 8th passages, p8) of the cultured astrocytes from 22-month-old aged mice brains. Scale bar, 50 μm. (**C**) Representative Western blot images showing EAAT-2, vimentin, and ALDH1L1 in young (Y) and aged (**A**) astrocytes (on 8th passages) cultured from 3-month-old and 22-month-old mice brains, respectively. (**D**) Flow cytometry analysis (fluorescence-activated cell sorting; FACS) showed different β-galactosidase levels between young and aged astrocytes in culture. Values represent β-galactosidase-positive cells among each population.

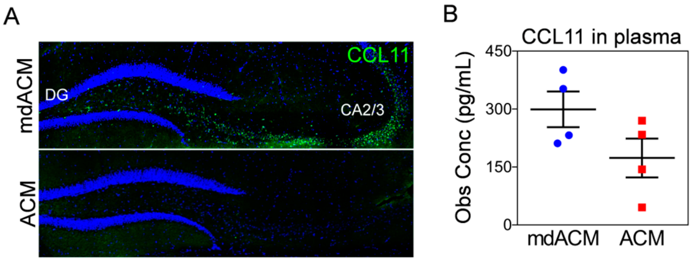

Supplementary Figure S2. Elevated CCL11 in the brain and plasma of aged mice are attenuated upon exposure to astrocytic Mt. (**A**) Representative immunohistochemistry showing reduced CCL11 staining (green) in the dentate gyrus (DG) and cornu ammonis (CA)2/3 area of hippocampus of aged (22-month-old) mice intravenously receiving the ACM-containing Mt as compared to animals receiving mdACM (Mt-depleted ACM). The animal received ACM or mdACM once a week for 4 weeks. Nuclei were stained with DAPI (blue). (**B**) Reduced CCL11 level in the plasma of aged (22-month-old) mice intravenously receiving ACM as compared to animals receiving mdACM. The animal received ACM or mdACM once a week for 4 weeks. CCL11 levels in the plasma were measured by BioPlex Luminex immunoassay. N = 4 animals per group. The significance was assessed by a two-tailed unpaired t-test: $p = 0.1141$, ACM vs. mdACM), t value ($t = 1.848$). All data are shown as mean ± SEM.

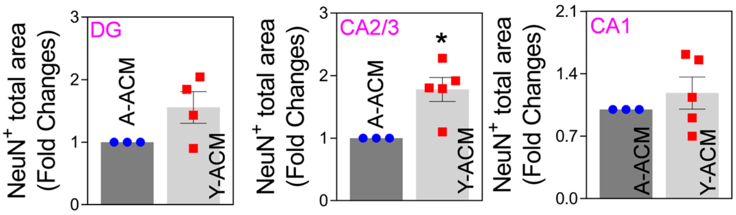

Supplementary Figure S3. The number of NeuN-positive cells in the hippocampus is higher in the aged mice that received young astrocytic Mt compared to aged mice that received aged Mt. Aged (22-month-old) mice intravenously received Y-ACM vs. A-ACM once a week for 4 weeks. By performing NeuN immunofluorescence staining in the hippocampal areas of aged (22-month-old) mice receiving Y-ACM vs. A-ACM, the $NeuN^+$ area in each brain was calculated. Three illustrating graphs show the difference in $NeuN^+$ area in each hippocampal sector, such as dentate gyrus (DG), cornu ammonis (CA)1, and CA2/3. The significance was assessed by a two-tailed unpaired t-test ($n = 3–5$ per group): * $p < 0.05$ ($p = 0.0223$, Y-ACM vs. A-ACM in CA2/3 area), t value ($t = 3.059$). All data are shown as mean ± SEM.
